# Regulation of Host Defense Peptide Synthesis by Polyphenols

**DOI:** 10.3390/antibiotics12040660

**Published:** 2023-03-28

**Authors:** Isabel Tobin, Guolong Zhang

**Affiliations:** Department of Animal and Food Sciences, Oklahoma State University, Stillwater, OK 74078, USA; isabel.tobin@okstate.edu

**Keywords:** host defense peptides, antimicrobial resistance, polyphenols, antibiotic alternatives

## Abstract

The rise of antimicrobial resistance has created an urgent need for antibiotic-alternative strategies for disease control and prevention. Host defense peptides (HDPs), which have both antimicrobial and immunomodulatory properties, are an important component of the innate immune system. A host-directed approach to stimulate the synthesis of endogenous HDPs has emerged as a promising solution to treat infections with a minimum risk for developing antimicrobial resistance. Among a diverse group of compounds that have been identified as inducers of HDP synthesis are polyphenols, which are naturally occurring secondary metabolites of plants characterized by the presence of multiple phenol units. In addition to their well-known antioxidant and anti-inflammatory activities, a variety of polyphenols have been shown to stimulate HDP synthesis across animal species. This review summarizes both the in vitro and in vivo evidence of polyphenols regulating HDP synthesis. The mechanisms by which polyphenols induce HDP gene expression are also discussed. Natural polyphenols warrant further investigation as potential antibiotic alternatives for the control and prevention of infectious diseases.

## 1. Introduction

Antimicrobial resistance has emerged as a major world health concern over past decades [1]. Excessive usage of antibiotics in human medicine and agriculture production has driven the emergence of resistant bacterial strains [1]. This issue has led to a significant increase in deaths associated with infections that were once treatable with antibiotics [1]. Development of novel antibiotics and antimicrobial approaches has become an intensive area of research [2]. Host defense peptides (HDPs), also known as antimicrobial peptides, elicit strong antimicrobial activity and regulate the immune response to protect against infections while minimizing inflammation [3,4].

Although synthetic and recombinant HDPs have shown clinical promise [5] and are also being used in livestock animals [6], direct administration of HDPs may be problematic due to the fact of poor pharmacokinetics, high production costs, and lack of efficacy in vivo [5]. Thus, the identification of small-molecule compounds capable of stimulating the synthesis of endogenous HDPs may provide a cost-effective, antibiotic-alternative approach to treat infections [7]. In fact, a number of HDP inducers have been identified and several have been proven effective in disease alleviation [7,8,9,10]. Recent evidence has shown that polyphenols, a large group of natural metabolites produced by plants, have the ability to upregulate HDP production with potential to mitigate antimicrobial resistance. This review is aimed at summarizing the current findings on polyphenols regulating HDP production and discussing their possible mechanisms of action.

## 2. Host Defense Peptides

The innate immune system, consisting of physical, chemical, and cellular barriers, serves as the host’s first line of defense against a wide variety of insults. As an important chemical barrier of the innate immune system, HDPs are small peptides (approximately 15–45 amino acids) found in virtually all organisms including mammals, birds, reptiles, amphibians, plants, and fungi [3,4,11].

### 2.1. Classifications of HDPs

Two major HDP families in vertebrate animals are cathelicidins and defensins [3,4]. Cathelicidins are produced abundantly in myeloid cells such as neutrophils and monocytes as well as skin keratinocytes and epithelial cells lining the respiratory, gastrointestinal, and urogenital tracts [12]. Synthesized initially as a precursor, cathelicidins are proteolytically cleaved from the cathelin pro-segment to become biologically active and released upon activation [12]. Defensins are characterized by the presence of six cysteine residues in defined positions [13]. Based on the location and spacing pattern of the cysteines, defensins are further divided into three subfamilies, namely α-, β-, and θ-defensins [13]. The α-defensins are produced abundantly by neutrophils and intestinal Paneth cells, while β-defensins are produced largely by the skin keratinocytes and epithelial cells of the gastrointestinal and urogenital tracts [13]. On the other hand, θ-defensins have a unique cyclic structure and have only been found in primates [14]. Like cathelicidins, α- and θ-defensins are translated as inactive precursors that are proteolytically cleaved at highly conserved sites to become mature active peptides to be stored in the granules of neutrophils or Paneth cells [14].

### 2.2. Mechanism of Antimicrobial Action of HDPs

**Figure 1:**
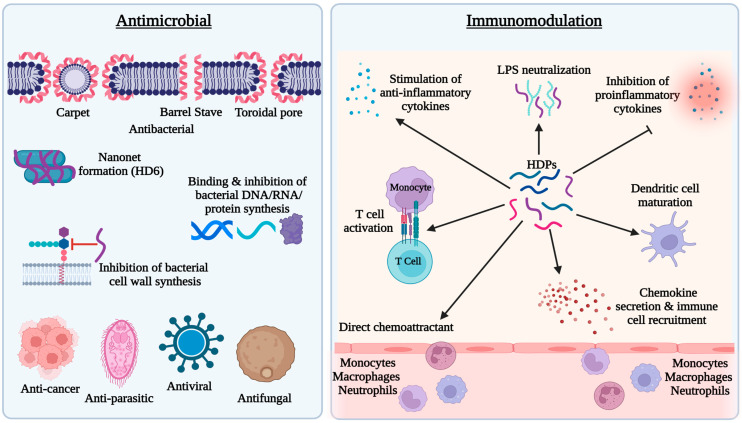
Antimicrobial and immunomodulatory activities of host defense peptides (HDPs). HDPs possess direct antimicrobial activity against bacteria, fungi, viruses, and parasites mainly through membrane disruption. A few HDPs also inhibit bacteria by forming nanonets or targeting bacterial DNA, RNA, proteins, or lipid II of the cell wall. Immunomodulatory effects of HDPs mainly include recruitment and activation of immune cells and regulation of inflammatory response.

HDPs are active against both Gram-positive and Gram-negative bacteria, fungi, parasites, and enveloped viruses, as well as cancer cells [3,4]. Several models, such as barrel stave, carpet, and toroidal pore, have been proposed to explain the antibacterial activity of HDPs [3,4] (Figure 1). The most accepted mechanism is based on the cationic and amphipathic natures of HDPs, which allow these peptides to interact with the outer membrane of bacteria composed of negatively charged phospholipid head groups. The cationicity of HDPs drives their attraction to the bacterial membrane causing disruption of the membrane structure leading to the leakage of cellular contents and bacterial lysis [3,4]. Additionally, other antibacterial mechanisms have been revealed with several HDPs (Figure 1). For example, human α-defensin 6 (HD6) forms a nanonet structure to entrap bacteria to prevent translocation into intestinal epithelium [15]. Proline-rich HDPs, such as indolicidin, kill bacteria by binding to intracellular targets such as DNA without membrane lysis [16]. Proline-arginine rich PR-39 directly inhibits or degrades proteins involved in DNA replication in the bacterial nucleus [17]. Furthermore, human β-defensin 3 (HBD3), has been shown to inhibit bacterial cell wall synthesis by interacting with the cell wall building block, lipid II [18].

### 2.3. Role of HDPs in Innate and Adaptive Immunity

In addition to direct antimicrobial activity, HDPs possess immunomodulatory activities [3,12] (Figure 1). HDPs are capable of directly recruiting different types of immune cells to the site of infection or indirectly through stimulation of chemokine production. For example, human cathelicidin LL-37 is chemotactic for neutrophils, monocytes, and T lymphocytes through engaging formyl peptide receptor 2 [19], while human β-defensins such as HBD2 and HBD3 chemoattract neutrophils and monocytes through interacting with chemokine receptor CCR2 [20] and they recruit dendritic cells and T cells through CCR6 [21]. Additionally, HDPs such as LL-37 and HBD3 can induce the expression of a variety of chemokines, such as CCL2/monocyte chemoattractant protein-1, CCL3/macrophage inflammatory protein-1α, CCL4/macrophage inflammatory protein-1β, CXCL1/Gro-α, and CCL22/macrophage-derived chemokine, which attract monocytes, macrophages, neutrophils, dendritic cells, and T cells subsequently to the sites of inflammation [22]. In addition to chemoattraction, many HDPs further induce maturation and activation of the cells involved in innate and adaptive immunity. For example, β-defensins induce the expression of co-stimulatory molecules, CD80, CD86, and CD40, on monocytes and dendritic cells in a Toll-like receptor-dependent manner [23,24].

Although they are capable of eliciting proinflammatory and immune responses to facilitate the clearance of pathogens, HDPs mediate largely anti-inflammatory responses during inflammation and infection by inhibiting the expression of proinflammatory cytokines such as interleukin (IL)-1β, IL-6, and tumor necrosis factor-α (TNF-α), while stimulating the production of anti-inflammatory cytokines such as IL-10 [25,26] (Figure 1). Furthermore, cationic HDPs also bind to anionic lipopolysaccharides (LPS) to neutralize its effect on inflammation [3,12].

### 2.4. Transcriptional Regulation of HDPs

As an important component of innate defense, certain HDPs such as human α-defensins and HBD1 are constitutively produced, while other HDPs like HBD2, HBD3, and HBD4 are induced upon infection and injury [27,28]. Pathogen-associated molecular patterns (PAMPs), such as LPS, bacterial DNA, and flagellin, can induce the expression of HDPs [27]. For example, *Psuedomonas aeruginosa* rhamnolipids act as a PAMP to activate transcription factors, such as nuclear factor κB (NF-κB) and activator protein-1 (AP-1), causing induction of *HBD2* in keratinocytes [29,30]. Proinflammatory cytokines such as TNF-α and IL-1α are known to induce the expression of HDPs such as HBD2 [31].

Additionally, a diverse group of small-molecule compounds have been shown to induce expression of HDPs [7,8,9,10]. Enhancing HDP synthesis by short-chain fatty acids (SCFAs) such as acetate, propionate, and butyrate has been well documented [10]. Medium-chain fatty acids are also capable of regulating HDP synthesis, albeit with reduced efficacy [32,33]. Amino acids such as L-arginine, L-isoleucine, leucine, and valine can induce β-defensin synthesis in the intestinal epithelium [34]. Histone deacetylase (HDAC) inhibitors, such as entinostat, valproic acid, and trichostatin, are very effective at upregulating HDP synthesis [9]. Other compounds such as vitamin D_3_, zinc, β-glucan, fructan, and lactose are also capable of inducing HDP synthesis [8]. Furthermore, probiotic bacteria such as *Lactobacillus* strains have the ability to upregulate *β*-defensin synthesis as well [35]. Recent development of several high-throughput screening assays has led to identification of a number of HDP-inducing compounds and their efficacy in disease control and prevention is being characterized [36,37,38,39,40,41].

## 3. Polyphenols

### 3.1. Classifications of Polyphenols

**Figure 2:**
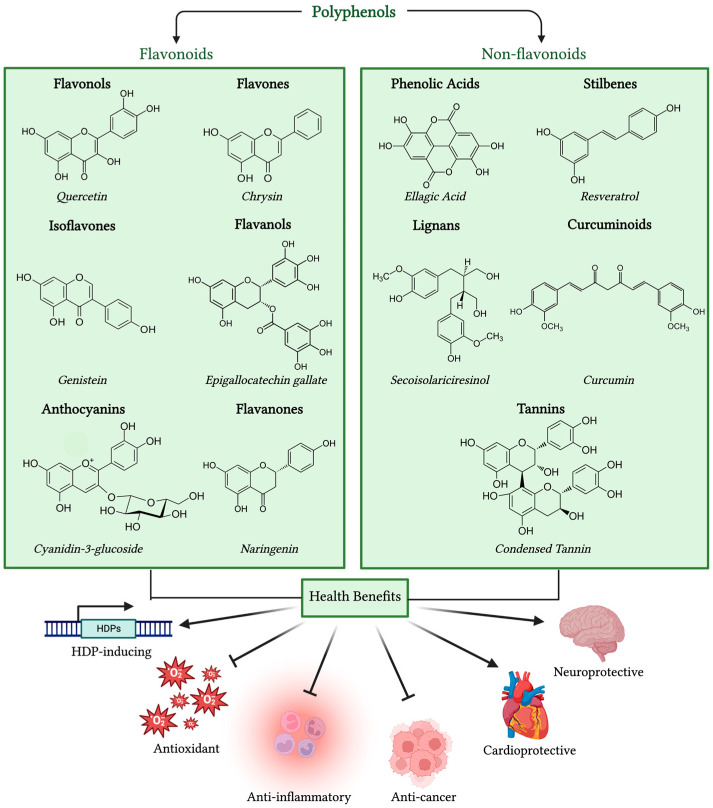
Classifications and health benefits of polyphenols. One representative compound is shown in each category for illustrative purposes.

Polyphenols are secondary metabolites produced abundantly in fruits, vegetables, tea, coffee, wine, and nuts as a mechanism of defense against pathogen invasion [42]. Polyphenols are structurally diverse, but share a foundational structure consisting of an aromatic ring with multiple hydroxyl groups [42]. Based on the structure, polyphenols are broadly divided into flavonoids and non-flavonoids. Flavonoids are further divided into six subcategories, namely, flavonols, flavones, isoflavones, flavanones, anthocyanidins, and flavanols, while non-flavonoid polyphenols mainly include phenolic acids, stilbenes, lignans, tannins, and curcuminoids [43] (Figure 2).

### 3.2. Antioxidant and Anti-Inflammatory Activities of Polyphenols

Polyphenols are well-known natural antioxidants and anti-inflammatory agents [42,43,44]. They act as scavengers of free radicals owing to the presence of an aromatic structure and multiple hydroxyl groups [44]. Polyphenols also attenuate oxidative stress by inhibiting enzymes involved in oxidation, such as NADPH oxidase, nitric oxide synthase, and xanthine oxidase [44]. On the other hand, polyphenols suppress inflammation mainly through inhibition of NF-κB, the arachidonic acid signaling pathway, and the mitogen-activated protein kinase (MAPK) pathway [45]. For example, resveratrol and curcumin alleviate oxidative stress by inhibiting NADPH oxidase activity [46,47], while quercetin inhibits the synthesis of proinflammatory cytokines in murine intestinal epithelial cells by inhibiting recruitment of NF-κB to target gene promoters [48].

### 3.3. Role of Polyphenols in Cancer, Cardiovascular, and Neurological Diseases

Polyphenols are also known to have anticancer activities and improve cardiovascular, metabolic, and neuronal health as supported by multiple epidemiological studies [49,50,51]. Modifications of key signaling intermediates involved in cancer-driving pathways such as PI3K, Akt, mTOR, RAS, MAPK, and p53 are suggested to be the main anticancer mechanism of polyphenols [49,52]. A possible explanation for polyphenol-mediated cardiovascular benefits is likely due to the fact that polyphenols are strong inhibitors of low-density lipoprotein oxidation, thereby reducing the risk of developing atherosclerosis [42]. Additionally, antioxidant and anti-inflammatory effects of polyphenols, together with their ability to enhance high-density lipoprotein levels and endothelial functions, also contribute to the cardio-protective property of polyphenols [42,50]. Similarly, the strong antioxidant effects of polyphenols are the major contributor to their ability to slow or inhibit neurodegenerative processes [51,53]. Polyphenols have been shown to prevent the development of diseases, such as Parkinson’s disease and Alzheimer’s disease, though the inhibition of acetylcholinesterase activity and β-amyloid aggregation within the brain, processes that are intensified by oxidative stress [51,53].

## 4. Regulation of Host Defense Peptides by Polyphenols

A number of polyphenols have been shown with the capacity to enhance HDP expression at mRNA and/or protein levels both in vitro and in vivo across a number of animal species including humans, mice, pigs, chickens, fishes, and insects (Table 1).

### 4.1. Regulation of HDPs by Flavonols

Quercetin is capable of inducing the synthesis of HDPs in multiple animal species. Quercetin augmented the mRNA expression of hepcidin antimicrobial peptide (*HAMP*) by 4-fold in human HepG2 hepatocytes and enhanced *HAMP* mRNA expression by over 600-fold in the liver of rats when injected intraperitoneally [54]. Zebrafish supplemented with quercetin had a significant increase in mRNA expression of defensin and liver-expressed antimicrobial peptide (*LEAP2*) in the liver [55]. Additionally, defensin and cecropin A mRNA expression was enhanced in the larvae of *Bombyx mori*, a silkworm, in response to dietary supplementation of quercetin [56]. Likewise, quercetin promoted the expression of multiple avian β-defensin (*AvBD*) genes in chicken HTC macrophage cells, further showing a strong synergy with butyrate [57]. Moreover, dietary supplementation of quercetin upregulated the expression of multiple *AvBD* genes in the chicken ileum [58].

### 4.2. Regulation of HDPs by Flavones

Flavone induced human cathelicidin (*CAMP*) expression in SW620 colon epithelial cells [59]. Similarly, chrysin enhanced *CAMP* expression by approximately 100-fold in A549 cells, a human lung epithelial cell line [60]. Saponarin, a flavone glucoside, also significantly increased *CAMP* expression, but decreased *DEFB1* expression in human HaCaT keratinocytes [61]. Apigenin, an active compound of chrysanthemums, promoted *DEFB1*, *DEFB2*, *DEFB3*, and *CAMP* mRNA expression in human epidermal keratinocytes [62]. Further, mice treated topically with apigenin for 9 days increased the expression of CAMP and MBD3 proteins, which was accompanied with increased production of lamellar bodies in the epidermis of mice [63]. Interestingly, flavones appear to play an opposite role during inflammation by downregulating HDP expression. For example, chrysin significantly decreased mRNA expression of *CAMP*, *DEFB2*, *S100A7* (psoriasin), *S100A8* (calprotectin A), and *S100A9* (calprotectin B) in human NHEK epidermal keratinocytes in response to stimulation with proinflammatory cytokines TNF-α, IL-17A, or IL-22 [64].

### 4.3. Regulation of HDPs by Isoflavones

Genistein, a soy isoflavone, upregulated *DEFB1* expression by approximately 30-fold in human LNCaP prostate cancer cell line [65] and also increased *CAMP* expression by 3-fold in human HaCaT keratinocytes [66]. Similarly, genistein and daidzein, another soy isoflavone, were found to promote the expression of porcine β-defensin 2 (*PBD2*) and *PBD3* in endometrial epithelial cells [67] and increase secretion of PBD1 and PBD2 in endometrial tissues [68]. Genistein- and daidzein-mediated induction of *PBD2* and *PBD3* was further enhanced in porcine endometrial epithelial cells treated with polyinosinic:polycytidylic acid (Poly (I:C)) [67], suggesting the possibility of these polyphenols to further stimulate HDP production during viral infections. Calycosin, an isoflavone found in the root of *Astragalus membranaceus*, triggered the expression of multiple porcine HDP genes, *PBD2*, *PEP2C*, and *PG1-5*, in 3D4/31 alveolar macrophages [39]. Moreover, calycosin stimulated *PBD2*, *PBD3*, and *PEP2C* expression in porcine IPEC-J2 cells and also enhanced *PBD3* expression in jejunal explants [39].

### 4.4. Regulation of HDPs by Flavanols

Epigallocatechin gallate (EGCG), a flavanol of green tea, and theaflavins, flavanols of black tea, significantly enhanced mRNA expression and protein secretion of HBD1 and HBD2 in human gingival epithelial cells and oral epithelial cells, respectively [69,70]. EGCG also induced mRNA and protein expression of HBD3 in bronchial epithelial cells, leading to an enhanced activity against H1N1 influenza virus [71]. In the same study, silencing the *DEFB3* gene abolished the antiviral effect of EGCG, indicating that EGCG-induced upregulation of HBD3 synthesis is essential for the antiviral activity of EGCG [71]. Additionally, EGCG induced *AvBD9* mRNA expression by 50-fold in chicken peripheral blood mononuclear cells (PBMCs) and further synergized with butyrate to induce *AvBD9* expression by over 3500-fold [57]. In porcine jejunal epithelial cells, EGCG promoted *PBD1* and *PBD2* mRNA and protein expression in a dose-dependent manner [72]. Moreover, EGCG reversed suppression of the mRNA expression of two human α-defensins, HD5 and HD6, in human Caco-2 colonic epithelial cells treated with Tegafur, an oral anticancer drug [73].

### 4.5. Regulation of HDPs by Anthocyanins

Anthocyanins such as cyanidin 3-O-glucoside increased total β-defensin protein levels in the intestinal mucosa of mice in an ovalbumin-induced food allergy model [74]. A blueberry powder, high in anthocyanins, supplemented in a high fat diet of rats for 8 weeks significantly increased *Defb2* mRNA expression by 2-fold in the ileum compared to rats on a low-fat diet [75]. Mice fed a high fat diet supplemented with an anthocyanin-rich grape pomace extract for 8 weeks restored the expression of regenerating family member 3 gamma (*REG3G*) to levels similar to rats on a low-fat diet [76]. However, delphinidin, an anthocyanin abundantly in pigmented vegetables and fruits, suppressed the induction of *S100A7* and *S100A15* (koebnerisin), together with many other proinflammatory cytokines, in inflamed psoriatic keratinocytes [77].

### 4.6. Regulation of HDPs by Phenolic Acids

Ellagic acid, a hydroxybenzoic acid, induced both mRNA and protein expression levels of HBD2 in primary human gingival epithelial cells [78]. Oral administration of caffeic acid phenethyl ester (CAPE) increased the mRNA expression of *Defb3* in mice by nearly 10-fold and reduced the colonization of *Candida albicans* in the oral cavity [79]. In the same study, CAPE also enhanced the expression of two antifungal defensins, galiomycin and galeriomycin, in the larvae of the greater wax moth *Galleria mellonella* [79]. Anacardic acid, a phenolic acid, drastically induced *AvBD9* mRNA expression by 100-fold in chicken PBMCs and further synergized with sodium butyrate to induce *AvBD9* mRNA expression over 1600-fold [57].

### 4.7. Regulation of HDPs by Stilbenes

Resveratrol, a well-known stilbenoid of grapes, has been found to regulate HDP expression both in vitro and in vivo across animal species. For example, resveratrol induced *CAMP* mRNA and protein expression in human U937 monocytes, further showing a strong synergy with 1,25-dihydroxyvitamin D_3_ [80]. Resveratrol caused a significant increase in murine *CAMP* mRNA and protein expression in both cultured human keratinocytes and mouse skin and further blocked invasion of *Staphylococcus aureus* into mouse skin [81]. Similarly, dietary supplementation of resveratrol enhanced mRNA expression of *Defa3*, *Defa5*, and *Defa20* in the ileum of mice [82]. Resveratrol also induced *HAMP* mRNA expression in liver cells by 4-fold as well as an 8-fold mRNA increase in the liver of rats [54]. Additionally, resveratrol upregulated *AvBD9* mRNA expression and also synergized with sodium butyrate to induce *AvBD9* expression in chicken PBMCs [57]. While resveratrol further upregulated *Streptococcus pneumonia*-induced expression of *DEFB2* in human A549 lung epithelial cells [83], the opposite occurred in A549 cells infected with *Pseudomonas aeruginosa* or nontypeable *Haemophilus influenzae* [84,85]. Pterostilbene and pinostilbene, a metabolite of pterostilbene, promoted the expression of *PBD3* and *PG1-5* in porcine 3D4/31 alveolar macrophages [39]. Isorhapontigenin, a methoxylated stilbene analog, was found to induce *PBD3*, *PEP2C*, and *PG1-5* mRNA expression in porcine IPEC-J2 intestinal epithelial cells and porcine jejunal explants [39].

### 4.8. Regulation of HDPs by Other Polyphenols

Several other classes of polyphenols have also been shown to regulate HDP expression. For example, curcumin, a curcuminoid isolated from turmeric, was found to cause a 3-fold induction of *CAMP* mRNA in human U937 monocytes and HT-29 intestinal epithelial cells [86]. Dietary supplementation of curcumin upregulated the mRNA expression of *DEFB1*, *HAMP*, and *LEAP2* in the liver and blood and also improved the survival of grass carp infected with *Aeromonas hydrophilla* [87]. However, condensed tannins and gossypol polyphenols were shown to downregulate *DEFB1*, *HAMP*, *LEAP2A*, and *LEAP2B* mRNA expression in the intestine and exacerbate *A. hydrophila* infection in grass carp [88,89]. Naringenin, a flavanone, promoted the expression of *HAMP* in HepG2 cells [54]. Chalcone polyphenol, isoliquiritigenin, induced *DEFB3* mRNA expression by 30-fold in human colonic epithelial cells [90]. More than 20 polyphenolic compounds were recently identified from a high-throughput screening with activity to induce *PBD3* expression in porcine IPEC-J2 cells [39]. Of those identified, xanthohumol and deoxyshikonin were revealed to be among the most potent inducers of *PBD3* in porcine IPEC-J2 intestinal epithelial cells, 3D4/31 alveolar macrophages, and jejunal explants [39].

Several studies have also revealed the benefits of polyphenol-rich foods, rather than an individual polyphenol, on HDP regulation. For instance, supplementation of pinto beans, which contain high amounts of phenolic compounds such as kaempferol, *p*-coumaric, gallic acid, vanilic acid, and *trans*-cinnamic acid [91], caused a 5-fold induction in *REG3B* and 2-fold increase in *REG3G* mRNA expression in the ileum of mice [92]. Dietary supplementation of another polyphenol-rich food, gold kiwifruit, also increased colonic expression of *Defb1* and *Defb2*, as well as improved barrier junction in mice [93].

**Table 1:** In vitro and in vivo regulation of host defense peptide expression by polyphenols.

Class	Polyphenol	Model	HDPs Affected ^1^	References
Flavonols	Quercetin	Human HepG2 hepatocytes	↑ *HAMP*	[54]
		Rat liver	↑ *HAMP*	[54]
		Zebrafish liver	↑ defensin, *LEAP2*	[55]
		Silkworm larvae	↑ defensin and cecropin A	[56]
		Chicken HTC macrophages	↑ *AvBD1*, *AvBD2*, *AvBD3*, *AvBD4*, *AvBD5*, *AvBD6*, *AvBD7*, *AvBD9*, *AvBD14*	[57]
		Chicken ileum	↑ *AvBD3*, *AvBD4*, *AvBD6*, *AvBD7*, *AvBD9*, *AvBD10*, *AvBD11*	[58]
Flavones	Flavone	Human SW620 colon epithelial	↑ *CAMP*	[59]
	Chrysin	Human A549 lung epithelial	↑ *CAMP*	[60]
		Human keratinocytes stimulated with TNF-α, IL-17A, or IL-22	↓ *S100A7*, *S100A8*, *S100A9*, *DEFB2*, *CAMP*	[64]
	Saponarin	Human HaCaT keratinocytes	↑ *CAMP*, ↓ *DEFB1*	[61]
	Apigenin	Human HaCaT keratinocytes	↑ *DEFB1*, *DEFB2*, *DEFB3*, *CAMP*	[62]
		Mouse epidermis	↑ *Camp*, *Defb3*	[63]
Isoflavones	Genistein	Human LNCaP prostate cancer	↑ *DEFB1*	[65]
		Human HaCaT keratinocytes	↑ *CAMP*	[66]
		Porcine endometrial epithelial	↑ *PBD2*, *PBD3*	[67,68]
	Daidzein	Porcine endometrial epithelial	↑ *PBD2*, *PBD3*	[67,68]
	Calycosin	Porcine 3D4/31 macrophages	↑ *PBD2*, *PEP2C*, *PG1-5*	[39]
		Porcine IPEC-J2 intestinal epithelial	↑ *PBD2*, *PEP2C*, *PBD3*	[39]
		Porcine jejunal explants	↑ *PBD3*	[39]
Flavanols	Epigallocatechin gallate	Human B11 oral epithelial	↑ *DEFB1*, *DEFB2*	[69]
		Human OBA-9 gingival epithelial	↑ *DEFB1*, *DEFB2*	[70]
		BEAS-2B bronchial epithelial	↑ *DEFB3*	[71]
		Chicken PBMC	↑ *AvBD9*	[57]
		Porcine IPEC-J2 intestinal	↑ *PBD1*, *PBD2*	[72]
		Human Caco-2 colonic epithelial stimulated with Tegafur	↑ *DEFA5*, *DEFA6*	[73]
Antho- cyanins	Cyanidin 3-O-glycoside	Mouse intestinal mucosa	↑ total β-defensin protein	[74]
	Blueberry powder	Rat ileum	↑ *Defb2*	[75]
	Grape pomace extract	Mouse colon	↑ *REG3G*	[76]
	Delphinidin	Human psoriatic keratinocytes	↓ *S100A7*, *S100A15*	[77]
Phenolic Acids	Ellagic acid	Human gingival epithelial	↑ *DEFB2*	[78]
	Caffeic acid phenethyl ester	Mouse tongue	↑ *Defb3*	[79]
		*Galleria mellonella* larvae	↑ galiomycin, galeriomycin	[79]
	Anacardic acid	Chicken PBMC	↑ *AvBD9*	[57]
Stilbenes	Resveratrol	Human U937 monocytes	↑ *CAMP*	[80]
		Human HaCaT keratinocytes	↑ *CAMP*	[80]
		Mouse epidermis	↑ *CAMP*	[81]
		Mouse ileum	↑ *Defa3*, *Defa5*, *Defa20*	[82]
		Human HepG2 hepatocytes	↑ *HAMP*	[54]
		Rat liver	↑ *HAMP*	[54]
		Chicken PBMC	↑ *AvBD9*	[57]
		*Streptococcus pneumonia*-infected human A549 lung epithelial	↑ *DEFB2*	[83]
		*Pseudomonas aeruginosa*-infected A549 lung epithelial	↓ *DEFB2*	[84,85]
	Pterostilbene	Porcine 3D4/31 macrophages	↑ *PBD3*, *PG1-5*	[39]
	Pinostilbene	Porcine 3D4/31 macrophages	↑ *PBD3*, *PG1-5*	[39]
	Isorhapontigenin	Porcine IPEC-J2 intestinal epithelial	↑ *PBD3*, *PEP2C*, *PG1-5*	[39]
		Porcine jejunal explants	↑ *PBD3*, *PG1-5*	[39]
Curcuminoid	Curcumin	Human U937 monocytes	↑ *CAMP*	[86]
		Human HT29 colonic epithelial	↑ *CAMP*	[86]
		Grass carp liver and blood	↑ *DEFB1*, *HAMP*, *LEAP2*	[87]
Tannins	Condensed tannins	Grass carp intestine	↓ *HAMP*, *LEAP2A*, *LEAP2B*, *DEFB1*	[88]
Flavanones	Naringenin	Human HepG2 hepatocytes	↑ *HAMP*	[54]
Chalcones	Isoliquiritigenin	Human Caco-2 colonic epithelial	↑ *DEFB3*	[90]
	Xanthohumol	Porcine IPEC-J2 intestinal	↑ *PEP2C*, *PBD3*, *PG1-5*	[39]
		Porcine 3D4/31 macrophages	↑ *PEP2C*, *PBD3*, *PG1-5*	[39]
		Porcine jejunal explants	↑ *PBD3*, *PG1-5*	[39]
Others	Gossypol	Grass carp intestine	↓ *HAMP*, *LEAP2B*, *DEFB1*	[89]
	Deoxyshikonin	Porcine IPEC-J2 intestinal epithelial Porcine 3D4/31 macrophages Porcine jejunal explants	↑ *PEP2C*, *PBD3*, *PG1-5* ↑ *PEP2C*, *PBD3*, *PG1-5* ↑ *PBD3*, *PG1-5*	[39] [39] [39]
	Pinto beans	Mouse ileum	↑ *REG3B*, *REG3G*	[92]
	Gold kiwifruit	Rat colon	↑ *Defb1*, *Defb2*	[93]

^1^ Abbreviations: HAMP, hepcidin antimicrobial peptide; AvBD, avian β-defensin; LEAP2, liver-expressed antimicrobial peptide 2; CAMP, cathelicidin antimicrobial peptide; DEFB, β-defensin; PBD, porcine β-defensin; DEFA, α-defensin; REG3, regenerating family member 3. “↑” denotes increased gene expression, while “↓” denotes decreased gene expression.

## 5. Mechanism of Action for Polyphenols

The mechanisms by which structurally diverse polyphenols regulate HDP expression are beginning to be revealed. The MAPK signaling pathway, the sphingosine-1-phopshate (S1P) pathway, the Kelch-like ECH-associated protein 1- nuclear factor erythroid 2-related factor 2 (KEAP1-NRF2) pathway, and epigenetics are involved in the regulation of certain HDP genes. In live animals, polyphenol-mediated alternations of intestinal bacterial metabolites are likely to contribute to enhanced HDP synthesis as well.

### 5.1. HDP Regulation by the MAPK Signaling Pathway

**Figure 3:**
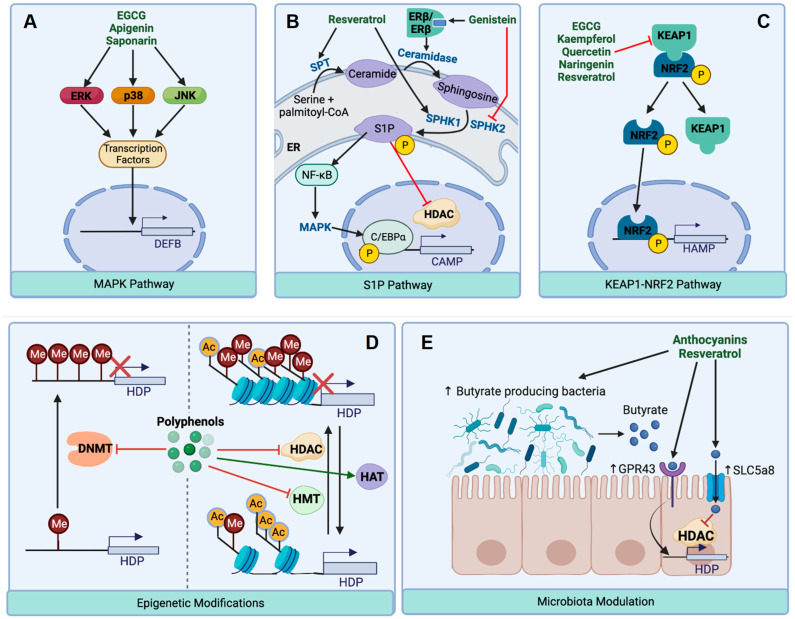
Molecular mechanisms of polyphenol-mediated induction of host defense peptide (HDP) genes. Certain polyphenols are shown to activate the MAPK pathway to upregulate the expression of human β-defensin (*DEFB*) genes (**A**). Resveratrol and genistein enhance sphingosine-1-phopshate (S1P) synthesis and NF-κB activation to induce cathelicidin antimicrobial peptide (*CAMP*) gene expression (**B**). Several polyphenols inhibit KEAP1 to allow nuclear translocation of NRF2 to induce the expression of hepcidin antimicrobial peptide (*HAMP*) (**C**). Many polyphenols function as inhibitors of histone deacetylase (HDAC), DNA methyltransferase (DNMT), or histone methyltransferase (HMT) to increase histone acetylation while reducing DNA and histone methylation to promote a relaxed chromatin structure in favor of HDP transcription (**D**). A few polyphenols directly enhance the histone acetyltransferase (HAT) activity to promote HDP transcription (**D**). HDP gene expression may be enhanced by polyphenols through modulating the synthesis of metabolites, such as butyrate, by the intestinal microbiota (**E**).

The MAPK signaling pathway is essential for a variety of cellular processes such as stress response and cell proliferation and differentiation [94]. The MAPK family consists of three major serine/threonine protein kinases namely, extracellular signal-regulated protein kinase 1/2 (ERK1/2), c-Jun N-terminal kinase (JNK), and p38 MAPK [94]. Once activated by external stimuli, these kinases will phosphorylate downstream proteins including transcription factors to regulate gene expression [94]. The MAPK pathways have been shown to be involved in polyphenol-mediated enhancement of HDP gene expression (Figure 3A). For example, EGCG-induced *DEFB1* and *DEFB2* expression is diminished in the presence of ERK/12 and p38 MAPK inhibitors in human gingival epithelial cells [69]. Similarly, EGCG enhances phosphorylation of ERK/12, JNK, and p38 MAPK in lung epithelial cells, while each of the three MAPK inhibitors suppresses EGCG-mediated *DEFB3* induction [71]. Induction of *PBD2* expression by EGCG is significantly reduced in porcine IPEC-J2 cells upon inhibition of p38 MAPK, but not ERK1/2 or JNK [72]. While they appear to activate the MAPK pathways in quiescent cells [69,71], polyphenols such as EGCG, apigenin, and saponarin clearly suppress the inflammatory response and the phosphorylation of ERK1/2, JNK, and p38 MAPK in cells during inflammation or infection [61,62,71].

### 5.2. HDP Regulation by the S1P Signaling Pathway

S1P is produced intracellularly from sphingosine on the cell membrane, acting as a signaling molecule involved in many cellular processes such as cell growth and cell trafficking [95]. Genistein-mediated increase in CAMP/LL-37 synthesis in keratinocytes is dependent upon estrogen receptor β (ERβ) [66] (Figure 3B). Engagement of genistein with ERβ induces the gene expression of ceramidase, which in turn stimulates S1P synthesis by hydrolyzing ceramide to form sphingosine. Increased S1P subsequently activates NF-κB and MAPK, leading to the phosphorylation of C/EBPα and initiation of *CAMP* gene expression [66]. Resveratrol has also shown to target the S1P pathway through activation of serine C-palmitoyltransferase (SPT) to stimulate S1P synthesis, resulting in NF-κB activation, C/EBPα phosphorylation, and enhanced *CAMP* gene expression [81]. Two sphingosine kinase isoforms, sphingosine kinase 1 (SPHK1) and SPHK2, synthesize S1P by phosphorylating sphingosine [95]. While S1P produced by SPHK1 stimulates *CAMP* gene expression, SIP generated by SPHK2 appears to be a negative regulator of *CAMP* expression [96]. It is not surprising that genistein specifically suppresses *SPHK2* mRNA expression [66], and resveratrol induces *SPHK1* mRNA expression [81].

### 5.3. HDP Regulation by the KEAP1-NRF2 Pathway

KEAP1 is a repressor of NRF2, a major transcription factor responsible for transcription of many genes with antioxidant properties [97]. Therefore, the KEAP1-NRF2 pathway plays a major role in regulating oxidative stress [97]. Polyphenols such as EGCG, kaempferol, naringenin, quercetin, and resveratrol activate the *HAMP* gene promotor by inhibiting KEAP1 to allow increased binding of NRF2 to the antioxidant response element (ARE) site of target gene promoters [54] (Figure 3C). Many other polyphenols such as curcumin and xanthohumol also activate NRF2 for enhanced antioxidant response [98,99]. However, whether NRF2 is involved in the ability of polyphenols to promote the transcription of other HDP genes remains to be investigated.

### 5.4. HDP Regulation through Epigenetic Modifications

#### 5.4.1. Inhibition of Histone Acetylation

Epigenetic modifications of DNA and histones play a very prominent role in gene expression [100]. Histone acetylation and methylation of DNA and histones are among the most common epigenetic modifications that alter chromatin accessibility and gene transcription [100,101]. Lysine residues in the histone tails can be modified by the addition or removal of acetyl groups by two opposing classes of enzymes known as histone acetyltransferases (HATs) and histone deacetylases (HDACs), respectively [102]. Increased histone acetylation is often associated with increased gene expression, while histone deacetylation is commonly associated with diminished gene expression [102]. Polyphenols such as resveratrol, quercetin, EGCG, genistein, and delphinidin are known to inhibit HDACs [103] (Figure 3D). For example, genistein increases the acetylation of histones H3 and H4 through enhancing HAT activity while suppressing HDAC activity [104]. EGCG has been shown to decrease the expression of proteins involved in heterochromatin formation such as HP1α and HP1γ as well as chromatin assembly factor 1A, thereby promoting a more relaxed chromatin structure [105], a condition favorable for HDP transcription. These observations are consistent with the fact that HDAC inhibitors are well-known HDP inducers [9]. In fact, S1P also inhibits HDAC1 and HDAC2 activity [106], which provides another explanation for increased S1P leading to enhanced HDP transcription.

Besides Zn-dependent classical HDACs, sirtuins are a family of NAD^+^-dependent HDACs [107]. Many polyphenols such as resveratrol, pterostilbene, quercetin, EGCG, and curcumin are sirtuin modulators [107]. Resveratrol further augmented *Streptococcus pneumonia*-induced *DEFB2* expression in human lung epithelial cells, while inhibition of sirtuin activity attenuated *S. pneumonia*-induced *DEFB2* [83], suggestive of the potential for natural polyphenolic compounds to modulate HDP synthesis.

#### 5.4.2. Inhibition of DNA and Histone Methylation

DNA methylation occurs mainly at cytosines by DNA methyltransferases (DNMTs), which leads to a condensed chromatin structure associated frequently with diminished gene transcription [101]. Methylation of the *DEFB1* promoter has been shown to cause a decrease in *DEFB1* mRNA and conversely, demethylation with a known DNMT inhibitor, 5-aza-2′-deoxycytidine, causes an increase in *DEFB1* transcription [108,109]. 5-Azacytidine and SGI-1027, two DNMT inhibitors, also induce *AvBD9* mRNA expression [110]. Polyphenols such as EGCG, quercetin, curcumin, and genistein are known to inhibit DNMTs [111,112], suggesting the possibility of these natural polyphenolic compounds to induce HDPs by acting as DNMT inhibitors (Figure 3D). Similar to DNA, histones can also be methylated to regulate gene expression [100,101]. Histones are methylated at the lysine or arginine residues by histone methyltransferases (HMT) [113]. Depending upon the position of lysine/arginine and the number of methyl groups to be transferred, histone methylation is associated with either transcriptional activation or repression [113]. Inhibition of histone methylation by specific inhibitors of G9a HMT (BIX01294) and EZH1/2 HMT (UNC1999) significantly enhanced chicken HDP gene expression in chicken HTC macrophage cells [110]. However, genistein increased histone H3 trimethylation at lysine 4 (H3K4me3) [104], a common marker of relaxed chromatin, which is consistent with the positive role of genistein in HDP expression [65,66].

### 5.5. HDP Regulation through Modulation of Gut Microbial Metabolites

Most dietary polyphenol intake is unabsorbed in the small intestine, thus the gut microbiota is critically important for biotransformation and metabolism of these products [114]. Polyphenol consumption is known to cause changes in the gut microbiota leading to alterations in bacterial metabolites such as SCFAs [114], which are well-known HDP inducers [7,8,9,10]. For example, polyphenol supplementation enriches butyrate-producing bacteria such as *Allobaculum*, *Roseburia*, *Butyricicoccus*, *Bifidobacterium*, and *Anaerotruncus* along with increasing SCFA production in mice [76,82], which may be partially responsible for polyphenol-mediated increases in HDP synthesis in live animals (Figure 3E). In addition to enhanced SCFA production, increased butyrate influx and signaling are likely to occur in vivo upon polyphenol supplementation. A dramatic increase in the mRNA expression of *SLC5a8*, a butyrate transporter, was observed in the colon of mice supplemented with polyphenol-rich extracts of cinnamon bark and grape pomace [76]. Supplementation of blueberries, rich in anthocyanins, caused an increase in mRNA expression of both *Defb2* and SCFA receptor, *Gpr43*, in mice [75]. GPR43 is in fact utilized by SCFAs to upregulate *REG3G* and *DEFB* expression in intestinal epithelial cells [115]. Therefore, polyphenol-induced SCFA synthesis by gut microbiota is likely to be involved in the in vivo induction of HDP synthesis.

## 6. Future Prospects and Conclusions

The use of HDPs as antibiotic alternatives to infectious disease control and prevention is promising due to the beneficial antimicrobial and immunomodulatory properties of these peptides [3,4,11]. Exogenous administration of HDPs have been found to be effective against various infections in both laboratory and livestock animals and a number of these peptides are in different stages of clinical trials [3,4,11]. However, because of high production cost and instability associated intrinsically with most peptide-based drugs, new technologies are being explored to improve HDP stability and efficacy such as nanotechnology-based formulation and delivery, peptide modifications, and non-peptide mimetics [3,11]. HDPs are also being tried as adjuvants to antibiotics and vaccines [3]. Alternatively, modulation of endogenous HDP synthesis is a promising host-directed approach to disease control and prevention in the age of antimicrobial resistance and a large number of natural and synthetic small-molecule compounds, such as polyphenols, have been found to be capable of inducing HDP synthesis both in vitro and in vivo [7,8,9,10].

Natural polyphenols are known to be antioxidant, anti-inflammatory, anticancer, cardio-protective, and neuro-protective. Current research further highlights the ability of polyphenols to boost HDP synthesis and innate defense, suggesting its potential for anti-infective application. Further studies on the in vivo efficacy of these polyphenols in different animal disease models are required to demonstrate the utility of these natural compounds as effective antibiotic alternatives. The MAPK signaling pathway, the S1P pathway, and the KEAP1-NRF2 pathway have been shown to be involved in polyphenol-mediated induction of certain HDP genes. In addition, epigenetic modifications such as histone acetylation, histone methylation, and DNA methylation by polyphenols play a critical role in HDP induction as well. Moreover, bacterial metabolites, SCFAs in particular, contribute to HDP gene induction in vivo upon polyphenol consumption. Further investigations on the mechanisms of action of these polyphenols are warranted to fully realize their potential in antimicrobial therapy.

Although polyphenols demonstrate many alluring health benefits, their use in human medicine has been limited due to the low oral bioavailability of many polyphenols [116]. Polyphenol absorption is inefficient due to chemical and physical characteristics causing low solubility and instability in the gastrointestinal tract [116]. On the other hand, polyphenols are known to be extensively metabolized by the intestinal microbiota and different metabolites of polyphenols exhibits various degrees of antioxidant and anti-inflammatory activities on the host [114]. However, it remains largely unknown how different polyphenol metabolites regulate HDP synthesis. It is perhaps more effective to use those metabolites that may demonstrate a superior HDP-inducing activity than the parental polyphenol compounds. It is also beneficial to encapsulate polyphenols or their metabolites for protection and extended release to the gastrointestinal tract. In fact, nanoencapsulation has improved absorption and bioavailability of polyphenols such as EGCG, curcumin, and resveratrol to increase their efficacy in vivo [117,118]. It is exciting to explore the potential of polyphenols as antibiotic alternatives to improve human and animal health while mitigating antimicrobial resistance.

## Data Availability

All data generated during this study are included in this published article.

## Abbreviations

AP-1, activator protein-1; ARE, antioxidant response element; AvBD, avian β-defensin; CAMP, cathelicidin antimicrobial peptide; CAPE, caffeic acid phenethyl ester; COX, cyclooxygenase; DEFA, human α-defensin; DEFB/HBD, human β-defensin; DNMT, DNA methyltransferase; EGCG, epigallocatechin gallate; ERK, extracellular signal-related protein kinase; HAMP, hepcidin antimicrobial peptide; HAT, histone acetyltransferase; HD5, human α-defensin 5; HD6, human α-defensin 6; HDAC, histone deacetylase; HDP, host defense peptides; HMT, histone methyltransferase; JNK, c-Jun N-terminal kinase; KEAP1, Kelch-like ECH-associated protein 1; LEAP2, liver-expressed antimicrobial peptide; LPS, lipopolysaccharide; MAPK, mitogen-activated protein kinase; MBD2, murine β-defensin 2; NF-ᴋB, nuclear factor ᴋB; NRF2, nuclear factor erythroid 2-related factor 2; NSAID, nonsteroidal anti-inflammatory drug; PAMP, pathogen-associated molecular pattern; PBD, porcine β-defensin; PBMCs, peripheral blood mononuclear cells; Poly (I:C), polyinosinic:polycytidylic acid; REG3, regenerating family member 3; REG3G, regenerating family member 3 gamma; S1P, sphingosine-1-phosphate; SCFA, short-chain fatty acid; SPHK, sphingosine kinase; SPT, serine C-palmitoyltransferase
